# Decision-making for Parents of Children With Medical Complexities: Activity Theory Analysis

**DOI:** 10.2196/31699

**Published:** 2022-01-17

**Authors:** Francine Buchanan, Claudia Lai, Eyal Cohen, Golda Milo-Manson, Aviv Shachak

**Affiliations:** 1 Institute of Health Policy, Management, and Evaluation University of Toronto Toronto, ON Canada; 2 Child Health Evaluative Sciences Research Institute The Hospital for Sick Children Toronto, ON Canada; 3 Department of Paediatrics University of Toronto Toronto, ON Canada; 4 Division of Developmental Pediatrics Holland Bloorview Kids Rehabilitation Hospital Toronto, ON Canada; 5 Faculty of Information University of Toronto Toronto, ON Canada

**Keywords:** shared decision-making, activity theory, parental decision-making, parenting, participatory medicine, pediatric, caregiving

## Abstract

**Background:**

Shared decision-making (SDM), a collaborative approach to reach decisional agreement, has been advocated as an ideal model of decision-making in the medical encounter. Frameworks for SDM have been developed largely from the clinical context of a competent adult patient facing a single medical problem, presented with multiple treatment options informed by a solid base of evidence. It is difficult to apply this model to the pediatric setting and children with medical complexity (CMC), specifically since parents of CMC often face a myriad of interconnected decisions with minimal evidence available on the multiple complex and co-existing chronic conditions. Thus, solutions that are developed based on the traditional model of SDM may not improve SDM practices for CMCs and may be a factor contributing to the low rate of SDM practiced with CMCs.

**Objective:**

The goal of our study was to address the gaps in the current approach to SDM for CMC by better understanding the decision-making activity among parents of CMCs and exploring what comprises their decision-making activity.

**Methods:**

We interviewed 12 participants using semistructured interviews based on activity theory. Participants identified as either a parent of a CMC or a CMC over the age of 18 years. Qualitative framework analysis and an activity theory framework were employed to understand the complexity of the decision-making process in context.

**Results:**

Parents of CMCs in our study made decisions based on a mental model of their child’s illness, informed by the activities of problem-solving, seeking understanding, obtaining tests and treatment, and caregiving. These findings suggest that the basis for parental choice and values, which are used in the decision-making activity, was developed by including activities that build concrete understanding and capture evidence to support their decisions.

**Conclusions:**

Our interviews with parents of CMCs suggest that we can address both the aims of each individual activity and the related outcomes (both intended and unintended) by viewing the decision-making activity as a combination of caregiving, problem-solving, and seeking activities. Clinicians could consider using this lens to focus decision-making discussions on integrating the child’s unique situation, the insights parents gain through their decision-making activity, and their clinical knowledge to enhance the understanding between parents and health care providers, beyond the narrow concept of parental values.

## Introduction

Children with medical complexity (CMC) are defined as individuals with complex chronic disease necessitating specialized care, high family-identified needs, functional disability, and high health care utilization [1,2]. As of 2007, it was estimated that CMC in Ontario, Canada comprised 10% of all hospital admissions and approximately one-quarter of hospital days [3]. The population of CMC is a heterogeneous one, including diverse medical conditions such as brain injuries, cerebral palsy, or extreme prematurity, conditions that are severe and complex due to the intersection of multiple organ systems being affected [4]. Due to their complex care needs, the demands on parents and families of CMC are high, with parents of CMC interacting, on average, with 13 different physicians and specialists representing 6 subspecialties [3]. In the United States, it is estimated that caregivers of CMC spend 11 hours to 20 hours per week coordinating the care their child receives from their multiple providers [5]. As a result, parents of CMC become intimately familiar with both the health care system and their child’s specific health care needs, as active participants in the provision of care.

Given the challenges of medical care for CMC, their parents are faced with many difficult decisions. Unlike parents of healthy children who seek occasional care for a broken bone or an acute respiratory illness, parents of CMCs often face a continuous number of interconnected decisions, often without the support of medical evidence due to the complexity and co-existence of multiple chronic conditions [6,7]. To support these complex decisions, shared decision-making (SDM), a collaborative approach to reach decisional agreement, is a proposed means of improving health outcomes for children with chronic medical conditions [8,9]. However, the application of SDM in pediatrics and for CMC specifically is still poorly understood [10] and underpracticed when compared with children without medical complexity [9].

Frameworks for SDM have been developed largely from the clinical context of a competent adult patient facing a single medical problem, presented with multiple treatment options informed by a solid base of evidence [7,10]. However, given that this is not the case for CMC, parents may undertake the decision-making process differently than adults facing a discrete medical choice. The goal of this study was to explore the decision-making of parents of CMC as an activity within the context of a process shared between clinician and parent but external of current SDM frameworks.

## Methods

### Overview

**Figure 1:**
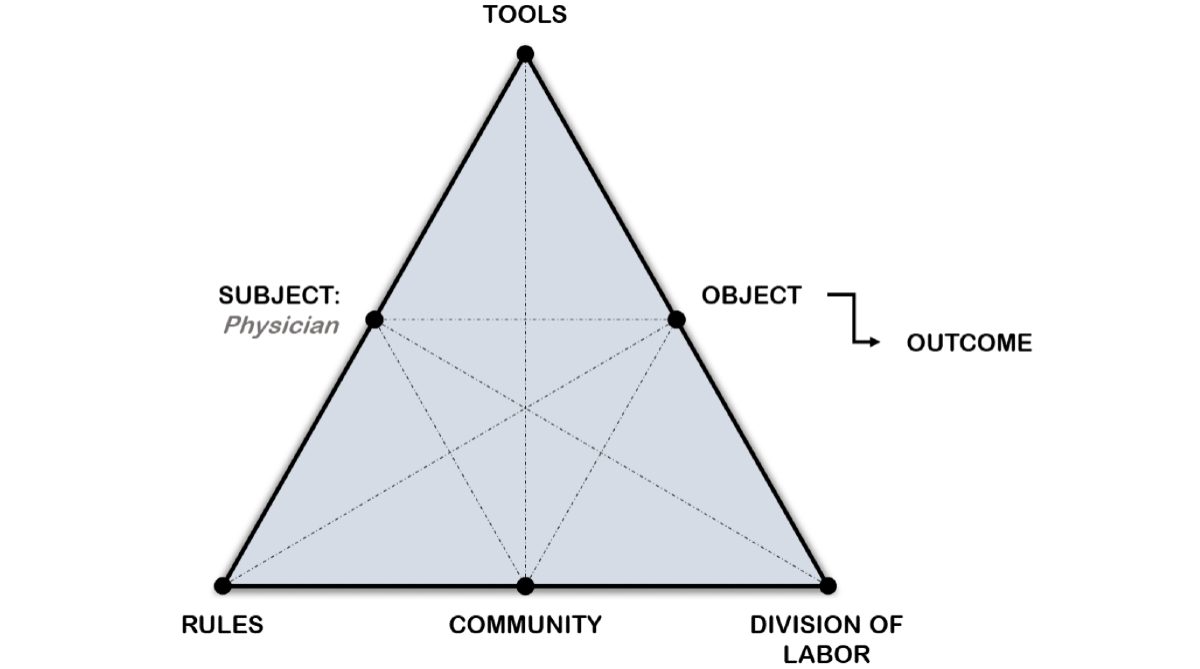
The activity system, adapted from [21].

In this qualitative study, activity theory informed both the data collection and analytical approaches taken. The semistructured interview method used in this study is based on the critical decision method (CDM) 5-step plan [11,12], supplemented with probes focused on the elements that comprise an activity as laid out in activity theory [13,14] using the Activity-Oriented Design Method (AODM) [15]. The interviews were analyzed using activity theory as a guiding framework and applying framework analysis methods [16-18]. A cross-disciplinary framework for studying different forms of human practices, activity theory provides a framework to view individual and social systems as interlinked, continuously evolving processes [19]. Activity theory was selected as the framework to guide both data collection and analysis as it provides a map that outlines the elements that comprise a human practice or activity considering an individual’s action, reactions, reasoning, and behavior with a broader context of influential rules, beliefs, and practicalities. The elements of the activity theory system as pictured in Figure 1 consist of (1) those involved in achieving the aim (Subjects), (2) the mediating artifacts used in the activity (Tools), (3) the rules that govern the activity (Rules), (4) other actors involved in the activity (Community), and (5) the division of activities among actors in the system (Division of Labor) [20]. The study was approved by the Research Ethics Board of the University of Toronto, and signed informed consent was obtained prior to each interview.

### Sample and Participant Recruitment

We recruited 12 participants (10 mothers, 1 father, and 1 young adult, formerly [child] with medical complexity) via social media groups for parents of children with medical needs in Ontario, Canada. The inclusion of a young adult CMC was a pragmatic decision, as the young adult joined the interview with their parent to elaborate on the story and provide additional insights. Eligibility criteria included being English-speaking, 18 years or older, and caring for a CMC. To determine if the child qualified as a CMC, each prospective parent participant completed a questionnaire listing the criteria for medical complexity (Multimedia Appendix 1). Parents who answered “yes” to at least 2 criteria for medical complexity were included in the study. Qualifying diagnosis for the CMC included children diagnosed with neurological disorders including cerebral palsy; rare diseases; and complex respiratory issues, including those requiring a tracheostomy and mechanical ventilation. Recruitment ended when thematic saturation was achieved. Saturation was reached when no new elements of the activity system, tools, rules, community, subject, object, and division of labor (Figure 1) were identified. The sample size is similar to studies utilizing similar methodologies [22,23].

### Data Collection

Data were collected using semistructured interviews based on CDM [11,12]. CDM is a type of cognitive task analysis interview and knowledge elicitation technique that consists of a 5-step semistructured interview plan with specific knowledge elicitation probes [12]. CDM’s 5 steps consist of (1) select incident, (2) obtain unstructured incident account, (3) construct incident timeline, (4) decision point identification, and (5) decision point probing. Interviews lasted 1 hour to 2 hours and were conducted in person by a single interviewer (FB). Participants were asked in advance to prepare a story about a time they had to make a difficult decision regarding the medical care of their child in consultation with their child’s medical team. The parent started the interview by relaying the story without interruption. To further elicit detailed information on “the judgements, assessments, and decisions” [11] along with the “motives, social and cultural issues within the context of the activity” [15], the interviewer used follow-up probes adapted from CDM [11] and the activity theory–informed AODM [15]. Notes were taken during the interview outlining the timeline of the decision, and keywords linked to each probe were documented. Interviews were also recorded and transcribed verbatim by a professional transcriber.

### Analysis

Interview transcripts and notes were analyzed using the 5 steps of the framework analysis approach developed by Ritchie and Spencer [16-18], namely familiarization, thematic analysis, indexing, charting, and mapping and interpretation. During the familiarization and thematic analysis phases, 2 researchers (FB, CL) independently read and open coded the same 3 transcripts looking for emergent themes and activity theory concepts (tools, community, rules, division of labor, object, and subject) [13]. A final coding scheme was developed by jointly discussing disagreements and reaching consensus on the themes and activity theory concepts identified in the data. At the indexing phase, the final coding scheme was used by the first author (FB) to re-code all 10 interviews in Nvivo 12 (QSR International; Burlington, MA). During the charting phase, relationships were established between codes, and similar codes were grouped together. Using Mwanza’s 8-step model [15] as a guide, the themes and activity theory elements were mapped to the activity triangles (Figure 1), and different activities were identified based on their objective (object within the activity system). Focusing on each activity and related subactivity as the unit of analysis, interpreting the data consisted of annotating the relationships between the elements that comprise the activity, noting the tensions, contradictions, and actions embarked upon to overcome them. The results of the analysis were sent to all interviewees to comment on the analysis and interpretation of the data as to further corroborate the findings. Feedback from interviewees included confirmation that the findings reflected their lived experience and suggestions to revised quotations that they believed would reveal their identity. Suggested changes were incorporated and approved by interviewees.

## Results

### Primary Interview Findings

The interviews conducted relayed stories about difficult decisions that ranged from the appropriateness of a surgical intervention to decisions around admission to hospital. Although the difficult decisions being discussed varied in terms of interventions, they were similar in that all were deliberated over multiple conversations with input from multiple health care providers. The decisions were also similar in that they all had a long-term goal of improving the child’s quality of life.

**Figure 2:**
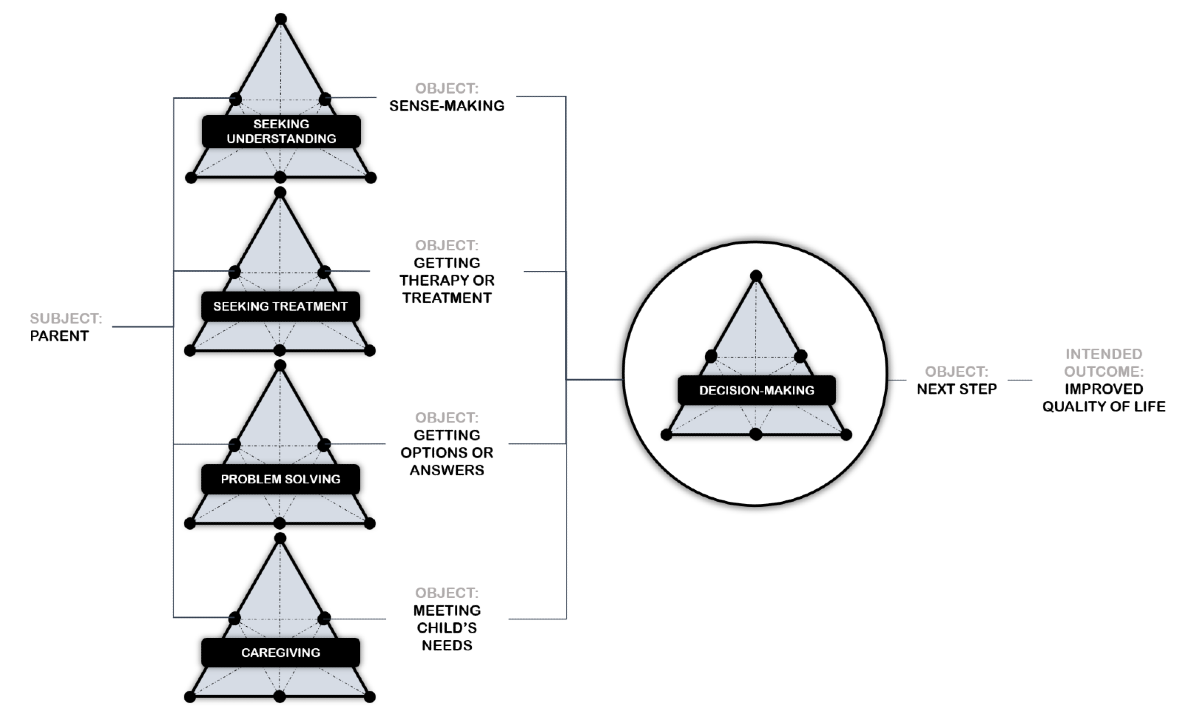
The parent’s decision-making activity system.

Our analysis of the parental decision-making process identified that the activity was comprised of 4 subactivities, outcomes of which were inputs into the larger decision-making process. Figure 2 depicts the relationship between the 4 activities of (1) seeking understanding, (2) seeking treatment, (3) problem-solving, and 4) caregiving and the larger decision-making activity. As noted in Figure 2, each subactivity was oriented toward different distinct immediate goals (object) that were necessary to achieve the outcome of the larger decision-making activity. For parents of CMC, decision-making was not just a single cognitive process of weighing the risks or choosing between available options. Rather, by engaging in the 4 subactivities, parents make sense of the context in which a decision is being made while also experimenting with problem-solving solutions to develop rules that govern and inform future decisions.

The narrative of our results in the following sections consists of describing how each subactivity unfolds, including an explanation of the actions taken by the parents (subjects) to overcome challenges that occur within the process.

### Subactivity 1: Seeking Understanding

The activity of seeking understanding was present in all the interviews and characterized by the need to seek out information to support the parents’ understanding of the situation they were facing. The aim (object) of this activity we label as “sense-making” as the activity is directed toward interpreting the situation as to transform it [24].

Because we really didn’t know. Like there is not a lot of information we were given. We kinda had to do our own research, figure it out and what not.

3002

The activity of seeking understanding is initiated by the parents being presented with a problem or decision point for which an answer was unclear.

We were presented with the idea after about two days after her heart surgery that we should try and extubate her and you know, not having I guess a full understanding of what that would mean for her or what that would look like because of the lack of knowledge around her lung and heart function.

3004

Parents engaged in the activity of seeking understanding when they found themselves unclear on how their child’s specific context could affect the outcome. For this activity, the subjects (parents) used a series of mediating tools, such as journal articles, test results, and Facebook to make sense of their child’s condition and formulate questions that, when answered, would improve their understanding.

These tools were validated against other cognitive and behavioral tools such as past experiences and inquiring questions. Access to health care providers in team meetings (community) also facilitated getting answers. The activity of seeking understanding involved questioning if the information being provided applied to their child’s specific needs.

The strategy going forward from there was to increase his medication, but they were doing it sooo slowly...I got around to actually looking at the literature myself on pediatric dosing, I was frustrated again because it was so low...the nurse who was doing the prescribing kept trying to, “He is on a lot of doses...I think he metabolizes it very quickly.” And the nurse had us try and go to the “normal” number of doses. Why? Why did she do that?

3006

Depending on the situation, the activity was focused on understanding why a health condition was occurring, why a specific suggestion was made, or the evidence to support a proposed course of action.

I want to understand things, I want to be spoke to in layman’s terms, I don’t like a lot of medical jargon that confuses me...I want them to dumb it down for me so I feel comfortable and I feel informed and I leave with the security of knowing that she’s going to...that I know. She may not get better, but I just need to be in the know.

3012

Activities of understanding were both successful and unsuccessful. Barriers that limited the subject’s ability to understand included gaps in the availability of information (tools), medical jargon, or systemic barriers to accessing the right people to answer the parents’ questions (rules). At times, these challenges led to an unfulfilled activity, resulting in uncertainty.

I don’t really know a lot about what the options are because with our last conversation, we didn’t really get a lot of information because we stopped the meeting because they realized that the key players were not in the room.

3011

A common barrier to understanding was not being involved in the discussions with doctors.

They [the doctors] weren’t involving us in any of their decisions. They were making decisions that we didn’t know that they were making without understanding the risks and benefits involved and without informing us of any risks that they understand that we didn’t understand...They would talk about whatever they would talk about, and they would come back with their decision, and we just weren’t involved.

3006

Not being involved in discussions left a gap in parents’ understanding of the reasoning and deliberations that led to a conclusion:

Just because someone tells you they have expertise does not mean that they’re using it properly and does not mean they have expertise in your child.

3006

The final decision or recommendation, even from doctors with extensive credentials, was not sufficient to support the parent’s ability to make sense of the situation.

When the activity was successful, the outcome was knowledge that informed further activities. When the activity was met with challenges or remained unsuccessful, those challenges were overcome by undertaking a secondary activity such as problem-solving or repeating the activity of seeking.

By making sense of their circumstance, parents felt more comfortable making a decision they felt was the correct one.

I didn’t have to think twice about it. You know, because everybody was already there, everybody gave their input. Here it’s like we get a little more information from this person but if they’re talking without the other person being there, so it’s like, would you say the same thing around the other person, right? So, I remember that was, when we made the decision, I made the decision by myself, I didn’t even tell my husband.

3002

In contrast, when families felt that they were blocked from gaining a full understanding of their CMC situation, they were unsure if the options and opinions put before them were the right ones. Parents wanted to understand why an option was put forward by clinicians, including the factors the clinicians considered and whether all available options were included in the deliberation.

I don’t know if we had exhausted all the measures to get the information that we needed around her heart.

3004

Gaps in the information or lack of appropriate tools to obtain a full understanding impeded the desire to conclude the decision-making activity.

### Subactivity 2: Seeking Treatment

The activity of seeking treatment is one where the parent either actively embarked on seeking out an intervention or passively agreed to the intervention suggested by the doctor and undertook the tasks to acquire it. The act of seeking out treatment took a large portion of each parent’s time, and much of that time was devoted to obtaining the treatment.

It wasn’t a difficult decision for us, it was difficult to get it to happen, it was difficult to get the doctors to decide to have it happen.

3006

Parents were driven to seek treatment or tests as an activity to obtain a solution to the identified problem. The outcomes of the activity were sought to provide input into a larger decision or as a tool to aid in the decision-making activity.

Parents developed their own set of tools to move the activity forward and overcome barriers. Persistence was a common tool utilized by parents in repeatedly engaging with health care providers. They often adapted their communication styles or the way they presented the situation based on how well the technique (or tool) has worked for them in the past:

I kept calling the secretary’s office, put us on a [surgery] cancellation list, put us on a cancellation list...Well I’ve come to learn sometimes you need to, uhmm, this is in air quotes “exaggerate the situation.” We said, well this isn’t really exaggerating but to the secretary it might have sounded [starts whispering] worse than it was.

3001

Taking on the role of advocates for their children, or as parents often framed it “I would push, I would push again and push harder” (3004), was what parents deemed necessary to overcome the barriers to accessing services, even though many parents did not want to take on such a role.

I didn’t like the position I was in, in that I had to tell the doctors to do their job. But I didn’t mind it, I had no qualms with telling them...

3012

However, when services were offered, parents felt more comfortable taking on more of a passive role within the division of labor.

They just sort of said, here’s the, this is the surgery, this is the surgery that he needs. And we at first said, okay, I guess if that’s the surgery he needs, that’s the surgery he needs.

3011

A common theme stated by parents was that they felt the need to trust their physician’s ability to balance evidence with the specific needs of the child. Parents were comfortable with taking a passive role only if they trusted that the options put forward were based on the specific needs and considerations of their child, after doctors have researched the full suite of options available.

Even so, our analysis did not identify that parents of CMC were aware of any tools that were used by doctors to convey their deliberation process. The result was a tension between the parents’ desire to trust the physician to execute their job (Division of Labor) and their need to validate that the physicians’ actions were based on the child’s specific needs and not other conflicting motives or influencing factors, including standard hospital rules or protocols.

So, I’m frustrated with the clinicians, why wouldn’t they tell you what are the options, do they not know these exist? I highly disbelieve that the neurologist who works at “Hospital A” doesn’t know about a gait lab, that her colleague runs. Why didn’t she tell me about this? ...Why won’t she say, hey, how about you go see a movement specialist? Do they not know, do they not want to tell us, are they overloaded and bombarded themselves that, you know, we’re just another number for them, they just want to move on to the next appointment?

3001

Access to available treatment options was an identified barrier dictated by the rules, community, and division of labor within the activity system. For example, rules requiring doctors’ referrals for certain procedures at times limited obtaining or changing therapy and treatment.

They fully said they will not do this procedure. At one point, I finally got to say, there is nothing I can do to change your mind on this?

3005

When the activity of seeking treatment was met with barriers, the outcome for parents was often frustration or uncertainty. For example, parents of CMC were frustrated that physicians controlled access to interventions because of the lack of reasoning provided.

They just said “no that is not how we do things.” That’s an exact quote. I will never ever forget it.

3006

The lack of information required to support decision-making drove parents to seek out other alternatives, such as embarking on a problem-solving activity of their own, repeating the seeking activity, or looking to understand the situation with insufficient tools (information). The barriers and facilitators identified in this activity informed how parents embarked on the decision-making activity or related activities. Parents looked to treatments available from accessible sources, such as accommodating physicians or peers:

Everything I asked for, she [the doctor] accommodated. Whereas sometimes if you ask a doctor for a certain test, they just disregard it and say they don’t need it. She was very open to ordering everything I asked for.

3010

When barriers arose, tools such as persistence were sometimes not enough, and luck often played an important role in gaining access to care.

I ran into our [specialist]...and she asked me how it was going, and I burst into tears (laughs) and then she helped us out. I don’t mean I like [made] a rational phone call and requested help. It was desperate times.

3006

The outcomes of this activity were not only the results of therapy but also knowledge of how the system’s rules work, development of the parents’ beliefs of their role, and knowledge on best sources of treatment options, which may not always be the physician.

This tacit knowledge of the system or observations from the therapy were integrated into the decision-making activity as best practices (tools), for example, always booking appointments with the same clinician to ensure consistency and continuity of care.

When we go to the clinic, we always schedule with the same orthoptist, we always schedule with the same ophthalmologist. That the vision clinic at “Hospital A” has like four or five different ophthalmologists, we specifically request the same clinicians so that, because their notes are consistent, they see the trend, they know, you know what I mean, instead of flip- flopping within the clinic.

3001

### Subactivity 3: Problem-solving

The activity of problem-solving is a process of trial and error, experimentation, and hypothesizing. Parents referred to the activity as their role or responsibility, which was required due to gaps in service provisions or for collecting evidence to support decision-making. The activity of problem-solving is oriented toward finding an answer to, or reason for, a specific problem with an aim toward achieving a longer-term goal (outcome) such as understanding options to present to clinicians.

The problem-solving activity is comprised of connecting and using tools such as test results, journal articles, social media, past personal experience, and observations.

We had worked really hard to learn as much as we could about the condition, to talk to our faith leaders, to talk to other parents, to talk to anybody and everybody we felt would be wise and to get as much of a sense of, like, we knew that we wouldn’t be able to answer all of the questions beforehand, but we wanted, like, a working theory on how we were going to answer the questions.

3005

Parents also reached out to community members such as peers, doctors, and family for assistance and insights.

Facebook, social media is how I learn everything.

3001

Social media provided parents with the opportunity to connect with peers who may also have experienced similar problems.

The activity was framed and influenced by rules such as the availability and access to tools or community members (clinicians or peers) willing to share their own experiences, which could be used as tools. Some parents referred to their own educational backgrounds as nurses, health care administrators, doctors, engineers, or basic scientists, which influenced how they viewed the problem but also provided them with skills to access and evaluate tools, such as journal articles. Having access to specific skills drove how they proceeded with the problem-solving activity but was also seen as something not visible or valued by health care providers.

I wish [health professionals] wouldn’t assume that all parents get their information from Google and Facebook, because, yes, obviously, I joined all the possible Facebook groups for parents of children with cerebral palsy and so on. But I also know how to use PubMed, I looked at, and I got my husband who is a doctor, and I got my husband to look at things with me. I feel like I've done the academic research, but I've also done the parent perspective side, because when you go on Facebook groups, people talk about these things like SDR surgery and what sorts of questions should I be asking, and what was your experience? I feel like I've covered both the real lived experience, and I also try to cover the academic evidence-based side. But health professionals always assume that parents just go on Facebook, or they say things like, well, stay off Google. Well, Google is not a bad starting point. It’s not somewhere you should necessarily end, but it’s not a bad starting point. I think health professionals actually need to give parents more credit because, yes, parents read everything, and a whole bunch of what they read might be irrelevant, but they also might read some stuff that’s valuable or relevant.

3007

In the problem-solving activity, the parents’ intended goal was sometimes fulfilled and at other times resulted in frustration when barriers were encountered. In one interview, the family explained how a limitation of access to medical equipment limited their ability to trial their solution, causing them to be frustrated as they attempted to get help from a clinician.

So, we kept thinking about it and trying to deal with it and we came up with this hypothesis that he was [health condition] and then we went to [the specialist] to ask if, we could maybe try [intervention] to see if [the intervention] would stop the [condition/symptoms].

3006

The influence of the rules or barriers resulted in outcomes that were at times different from the intended ones. This resulted in either frustration, uncertainty, or in gaining knowledge or experience. The resulting outcome drove the next activity such as seeking, caregiving, or making a decision with the newfound information or hypothesized solution.

### Subactivity 4: Caregiving

The activity of caregiving was represented in all 10 interviews. Participants described how they managed doctors’ appointments, delivered medical care, observed medical problems, and tried to keep their child happy and healthy. In the activity of caregiving, parents learned about their children, responded to their needs, and documented their progress. Like a detailed medical chart, some parents collected and collated years of data as part of the caregiver activity:

We had all the results there, we had all of the names of all the doctors there, we, I could give them [child’s name] birth weight, I could give them their weight at a year, I could give them their weight at two years, I could tell them every infection they had, like they couldn’t have asked for any more detail than we had.

3005

These parents did not embark consciously on a data collection activity. Rather, in the act of caregiving, they identified barriers to accessing data already collected in medical encounters and imbedded collection or collation of data as part of that activity. Similarly, gaps in training or knowledge were identified during the caregiving activity.

I realized that I could tell when he was having an apnea episode very easily and I could rub his back and that would get him breathing again. But like nobody explained to me any of these things.

3005

The act of caregiving was mediated by a variety of tools, such as access to information on how to provide proper care, personal observations, and knowledge gained from past experiences. The activity of caregiving is a continuous cycle of using tools to help decide what to do, observing the outcome, then re-examining the child’s condition to inform next steps. If the outcome was negative, the activity was reoriented to one of the related activities to obtain new knowledge and tools to continue the process.

Rules were a major driver of the activity of caregiving. The rules imposed by the child’s medical condition and treatment, such as specifically timed medications or use of a ventilator to maintain life support, all drove the act of caregiving, sometimes causing stress:

There was no leisure, there was no going and doing anything. And then the stress that we were under ...all the time was just crazy.

3006

These rules, motivated by medical needs, drove the need for caregiving tasks and restricted the ability to do the task but also drove the desire to find solutions to ease the burden of these tasks.

We had him vented 24/7 again...[but] we knew that it didn’t have to be this way...So we weren’t really like invested in figuring out how to move around with the vent. We were invested in getting him off the vent.

3006

In the act of caregiving, parents identified changes in their children’s needs but also identified gaps in how health care providers addressed those changes. Hospital rules that silo care and limit interdisciplinary and team-based care drove parents to take on the role of care coordination to overcome this barrier. Coordination was a role frequently cited in the interviews, as parents were able to view the full picture of the child’s care, whereas health care providers only saw pieces of it:

I’m the one who takes care of all her care. I’m the one who knows all of the moving pieces. I’m the one who is with her every day. Other parts of her team see her maybe once every few months. They don’t know the day-to-day of what she’s going through and what impact things will have on her.

3011

This kind of episodic care, born out of how hospitals are structured (rule), drove parents to act as coordinators of care. When the activity unfolded well, parents were happy that their child was living a fulfilled life. When the activity was met with barriers and outcomes were not achieved, it could cause frustration and uncertainty. Irrespective of how the activity unfolded, a secondary outcome of the caregiving activity was gaining experience and confidence but also a feeling that their expertise was not valued enough by health care professionals.

I know X [child name] at her best, I know X at her worst. I know X in-between. I’m able to gauge if the concern should be high. I am able to gauge if the concern should be low. I know X, I know every single thing about that little human being, that they just don’t know. They know [disease], but I know X’s [disease], and every kid’s [disease] is different. They sometimes, they’re just too stuck in their textbook definitions of what [disease] is, but X’s version of [disease] is what I know, and so that makes me an expert that they don’t give enough credit to.

3012

Outcomes of the caregiving activity were identifying decisions to be made, validating parents’ roles in the decision-making activity, and providing the knowledge to support the decision. The knowledge of a child’s reactions to medication, therapy, or treatments was an outcome of the caregiving activity, which became an input to the decision-making activity. From coordinating care, parents also collected, cross-validated, and documented information from multiple clinicians. This information then became a tool in the decision-making activity. In addition to tools, the caregiving activity provided parents with a sense of their role in the decision-making process and with confidence to make an informed decision or identify their knowledge gaps.

## Discussion

### Principal Findings

Frameworks of SDM in pediatrics are evolving. What was once viewed as the process of supporting a patient and their caregiver in choosing between multiple treatment options has now incorporated the understanding that the complex reality of making decisions is underserved “by depicting the making of ‘a’ decision as a discrete act” [7]. The findings of our study support recent findings from Feudtner et al [7] that decision-making by parents of CMCs consists of multiple decisions that shape and inform future care decisions. By using an activity theory lens, our study identified that parents of CMCs make decisions based on a mental model of their child’s illness, informed by the activities of problem-solving, seeking understanding, obtaining tests and treatment, and caregiving. Our findings depict the parental decision-making process as a continuous process connecting the parent’s past, with decisions made in the present and future.

Whereas previous studies have identified a multitude of influences affecting parental decision-making, including “cultural norms, community standards, impact on siblings or extended family, previous experiences, religious faith, and impact of acuity and stability of the child’s health status” [8,25], our study instead focused on how these background elements, combined with systemic rules and beliefs in the participants’ roles, drive actions and decisions. The activity theory framework and the probes developed from previous work completed by Hoffman et al [11] and Mwanza [15] focused the interview on identifying the needs and the activities undertaken to fulfil them. The framework of activity theory deconstructed the complexity of the decision-making task into smaller pieces (elements within the activity system) [13] that could be analyzed to determine the relationships between elements and how they evolve over time. Using activity theory as the structure for the data analysis not only organized the tasks that comprise the larger activity but also revealed the distinct short-term goals (objects) that informed the actions that parents embarked on, thereby informing the larger activity (eg, caregiving).

Proposed frameworks for SDM in pediatrics are still grounded in the belief that the goal of SDM is to improve medical outcomes for children by combining parental values with current evidence [8-10]. However, the findings of our study suggest that the basis for parental choice and values brought to the decision-making activity are developed via activities looking to build concrete understanding and capture evidence to support their decisions. What has been conceptualized as parental values in pediatric decision-making models are in fact tools developed from parents’ activities, which serve to support the larger decision-making activity. Parental beliefs and values described in SDM models are identified in our study as concrete tools that include findings from experimentation, behaviors learned from prior medical encounters, and observations gained from performing caregiving tasks.

These tools, which others have presented as heuristics, “ease the tasks of decision-making because they fit unfamiliar, complex, or novel information into familiar patterns of thought and language. By using common maxims and rules of thumb, parents can tackle the current challenges of decision-making by casting the daunting situation in terms and concepts that in the past have helped to make sense of other situations, solve problems, and communicate” [26]. Our study has taken steps to identify the activities that develop the heuristic tools identified by Renjilan and colleagues [26] and show that the activities that create them are an integral part of the decision-making activity.

The activities parents complete to formulate their decisions are important to understand in-depth when developing solutions to improve SDM for CMC. For example, in the act of problem-solving, parents formulate a hypothesis or potential solution to the problem that they bring with them to decision-making deliberations. However, without tools for doctors to explore these hypotheses and how they were formulated, these are often excluded from decision-making discussions and minimized to parents’ values. Additionally, our findings that parents embark on an activity to understand their child’s medical reality are key to addressing gaps in current SDM models. Acknowledging that parents make decisions based on an understanding that is constructed by engaging in concrete actions, rather than just developing abstract values, further supports the importance of parental contribution to the decision-making process as active participants. The findings of our study detail the specific activities performed by parents that build their sense of empowerment, expertise, and knowledge. An important next step in this area of work is further empowering parents with the knowledge that the activities they perform are important and valued in the SDM process. Practice recommendations outlined in Table 1 provide examples of how clinicians can support empowered SDM by incorporating the findings from this study into SDM conversations.

Our study has several limitations. First, this research focused on the decision-making practices of parents only and did not consider the perspectives of the physicians involved in the decision-making. Although the rich narratives we obtained provide insight into parent’s actions and reasoning for those actions, our study relied on retrospective accounts using a single data collection method. To mitigate the potential for recall bias, future research may apply additional methods, such as observations of parent-physician encounters in situ. Second, we chose to focus on CMC as it is a population with extraordinary health needs who are supported by caregivers that are generally highly invested in the health of their children. Thus, the perspectives of parents of CMC in our study may not be generalizable to parents of other pediatric populations.

**Table 1:** Practice recommendations for clinicians embarking on shared decision-making (SDM) for children with medical complexity (CMC).

Key findings	Practice recommendations
Parents make decisions based on their lived experience: Parents of CMC use information collected from the daily acts of care such as problem-solving, seeking understanding, obtaining tests and treatment, and caregiving to inform their decision-making. As active participants in the delivery of care, parents of CMC develop their expertise as caregivers and gain a valuable knowledge to inform decision-making.	Empower parents by acknowledging that the daily activities they perform in caring for their child are the basis for their expertise as caregivers and a valuable source of knowledge to inform decision-making. When seeking parental perspectives to inform SDM, direct questions toward parental knowledge, actions, and observations parents have made, rather than only their long-term goals or broad values they may hold.
Understanding is contextual: When trying to understand their child’s medical condition (sense making) parents endeavor to gain a sense of how their child’s specific context could affect the outcome. Parents want to trust that the options presented by the physician are based on the specific needs and considerations of their child.	When presenting medical options for care, provide background and reasoning in relation to the child’s specific needs, family context, the larger body of options considered, and known evidence base. Consider connecting parents with peer families to facilitate discussions that may address practical, social, and community issues grounded in lived experience.
Multiple activities influence decision-making: Parents make decisions based on the completion of multiple activities including caregiving, problem-solving, obtaining treatment, and sense making.	Be mindful of the needs of parents that may fall outside of immediate decision deliberation but still impact how decisions are made (eg, vacation time for parents considering a surgical intervention). Provide a supportive environment to discuss all aspects of care related to the decision-making process including the outcomes of, caregiving, problem-solving, obtaining treatment, and sense making. Consider tools and resources that can support the decision-making process outside of clinical encounters.
Rules guide and influence activity outcomes: Rules such as cost of therapy or medication can limit the number of options available to parents. Parents make decisions fully aware of these limitations.	Be aware of rules or structures that may be limiting the ability of parents to fulfill the options presented to them and address them openly (eg, presenting options that are too expensive).

### Conclusion

When viewing the decision-making activity as a combination of the caregiving, problem-solving, and seeking activities, we can address both the aims of each individual activity and the related outcomes (both intended and unintended). Understanding that the outcome of problem-solving is a carefully crafted idea or hypothesis should focus clinicians on questioning what occurred in the problem-solving activity to develop that idea. When addressing how to educate parents on the medical options, it could be useful to view the parents’ seeking understanding as a sense-making activity aimed at bridging the gap between their current situation, specific to their child’s personal context, and desired outcomes [27,28]. This view could help clinicians focus conversations toward integrating the child’s unique situation with knowledge gained from general standards of care and help reach greater understanding between parents and health care providers, beyond the narrow concept of patient (or parental) values. Challenging the belief that, in SDM deliberations, patients and families bring values and physicians bring clinical expertise, similar to other studies [29], our findings show that parents are active participants in the delivery of their child’s health care. Thus, viewing the information and insights gained from the caregiving, problem-solving, and seeking activities as broader than values should inform physicians to engage with the information provided by parents as a form of expertise.

## Appendix

Multimedia Appendix 1 Eligibility questionnaire for a parent of a child with medical complexity.

## Abbreviations

AODM: Activity-Oriented Design Method
CDM: critical decision method
CMC: children with medical complexity
SDM: shared decision-making
