# Supplementary material for: Decision-making for Parents of Children With Medical Complexities: Activity Theory Analysis
Source: J Particip Med. 2022 Jan 17;14(1):e31699. doi: 10.2196/31699 (PMC8804956; doi:10.2196/31699)
Supplement: Multimedia Appendix 1 [file jopm_v14i1e31699_app1.docx]

[**Multimedia Appendix 1**. Eligibility questionnaire for a parent of a child with medical complexity.](https://jopm.jmir.org/api/download?filename=56c77a77a3c1fd04f7d0d31f5ca9d22c.docx&alt_name=31699-579766-1-SP.docx)

**Parent of a Child with Medical Complexity Eligibility Questionnaire**

**Q1:** Are you currently a caregiver to a child with one or more disabilities? (Y/N)

**Q2:** Does the child in your care meet all of the following criteria:

- Has one or more chronic condition(s), either diagnosed or unknown.

AND

- Requires frequent and/or prolonged visits to the hospital, pediatrician, or therapist or the ongoing involvement of multiple subspecialty services and providers.

AND

- Is reliant on medical technology, assistive support person(s), or equipment to support daily activities (feeding tube, ventilator, bi-pap, tracheostomy, wheelchair, etc.)

**Q3:** Do you consider yourself fluent in English? (Y/N)

**Q4:** Has the child in your care received care at a local children’s hospital, children’s treatment center, or specialty pediatric clinic within the past 6 months? (Y/N)

**Q5:** Do you consider yourself primarily responsible for the health care–related choices regarding the child in your care? (Y/N)

**Q6:** Does the child in your care currently reside with you in your private residence? (Y/N)

**Q7:** Do you feel comfortable sharing a story about the child in your care receiving medical care? (Y/N)
